# Reflex Control of Robotic Gait Using Human Walking Data

**DOI:** 10.1371/journal.pone.0109959

**Published:** 2014-10-27

**Authors:** Catherine A. Macleod, Lin Meng, Bernard A. Conway, Bernd Porr

**Affiliations:** 1 Department of Biomedical Engineering, University of Strathclyde, Glasgow, Scotland; 2 Biomedical Engineering, School of Engineering, University of Glasgow, Glasgow, Scotland; University of Toronto, Canada

## Abstract

Control of human walking is not thoroughly understood, which has implications in developing suitable strategies for the retraining of a functional gait following neurological injuries such as spinal cord injury (SCI). Bipedal robots allow us to investigate simple elements of the complex nervous system to quantify their contribution to motor control. RunBot is a bipedal robot which operates through reflexes without using central pattern generators or trajectory planning algorithms. Ground contact information from the feet is used to activate motors in the legs, generating a gait cycle visually similar to that of humans. Rather than developing a more complicated biologically realistic neural system to control the robot's stepping, we have instead further simplified our model by measuring the correlation between heel contact and leg muscle activity (EMG) in human subjects during walking and from this data created filter functions transferring the sensory data into motor actions. Adaptive filtering was used to identify the unknown transfer functions which translate the contact information into muscle activation signals. Our results show a causal relationship between ground contact information from the heel and EMG, which allows us to create a minimal, linear, analogue control system for controlling walking. The derived transfer functions were applied to RunBot II as a proof of concept. The gait cycle produced was stable and controlled, which is a positive indication that the transfer functions have potential for use in the control of assistive devices for the retraining of an efficient and effective gait with potential applications in SCI rehabilitation.

## Introduction

Human walking can be viewed as a complex programme of reflexes which through the use of feedback and feed-forward processes allows stepping to adapt to a constantly changing terrain or walking environment. Loading and contact information from the feet are important sensory components in producing a walking pattern which is flexible and efficient, and can be measured directly or indirectly by a variety of specific and non-specific receptors which is then fed back to control the stepping. Gait is cyclical in nature with intrinsic muscle properties providing many constraints which can have an influence on individual muscle function and the coordination of multiple muscles to perform the locomotion [1]. The stability of human bipedal gait is due to a coherence between the body's neuromuscular skeletal system and the walking environment [2].

Many different control strategies have been used within robotics, not only to produce bipeds with a stable and efficient gait pattern, but also for studying biological models and gaining insight into walking control systems that may be present in humans. This allows us to simplify and analyse individual components of a complex system to study their role in generating functional locomotion. From this information, development can be made in the area of rehabilitation engineering with the aim of improving functional gait in individuals with spinal cord injuries (SCI) and other neurological injuries. Rehabilitation technologies for restoring ambulatory function and retraining of a functional gait include devices such as the exoskeleton, ReWalk (Argo Medical Technologies Inc., Marlborough, MA, USA) [3] and the robotic gait orthosis, Lokomat (Hocoma, Switzerland) [4]. Functional electrical stimulation (FES) has been demonstrated to be an important therapy, which can vastly improve walking function in individuals with incomplete spinal cord injuries (iSCI) [5]. FES is commonly recruited as a rehabilitation strategy for SCI to exercise and strengthen weakened muscles as well as artificially replace muscle activation that is missing or lacking (for review see [6]). However, high human energy requirements and a current complexity of FES systems for assisting walking mean these devices are not routinely used [7]. It is thus of fundamental importance to find a successful mechanism to control FES, one which is real-time, simple and does not override or counteract voluntary control originating from the user.

Classical control strategies employed in bipedal robotics, which have a biomechanic inspired design, include passive dynamic walkers, that are simple and can remain stable while walking down slopes [8]. Robots featuring this design have demonstrated gait, which appears visually human-like, however they cannot adapt and/or change their speed or walk on a level or inclined surface without the addition of actuators and controllers. Conversely, other robotic walkers, such as the well publicised bipedal walker ASIMO [9], have moved towards highly complex systems such as precise joint-angle control and trajectory-based methods (including Zero-Moment Point (ZMP) based [10] and Virtual Model control [11]). However the need for precision in the actuators and frequency response of these systems cannot be easily related to the human model which uses the less precise musculoskeletal system integrating muscles, tendons and joints under neuronal control [12]. Central pattern generator (CPG) methodology has also been investigated for creating humanoid bipedal robot walkers, which can be partially autonomous using local oscillators to generate limb motion patterns and limited sensory information as feedback (for review see [13]). Although this technique has proved successful in producing gait in a range of robotic walkers, including bipeds [14]–[16], and uses a biological approach conclusively described in animal locomotion, there remains debate over the importance of this strategy in human walking control. This has promoted development of biped locomotion controllers based on reflexes rather than on CPGs [17]–[19].

Within a human model, feedback on the current status of the walking process is fed back from different sensory organs located in muscles and tendons and from the peripheral vestibular and visual systems. At high walking speeds, coordination between the sensory input and motor output needs to act efficiently and quickly, which are high dynamic walking demands very difficult to replicate using existing biologically-inspired robotic control systems [15], [18], [20]. The gait cycle of bipedal walkers has only one foot in contact with the ground for the majority of the time, which is a major issue in the development of dynamic control avoiding tripping or falling. The development of RunBot I was able to demonstrate that minimal adaptive neuronal control, based on a reflexive mechanism [21] integrated with a biomechanic inspired design, can produce a fast walking and adaptive robot with a maximum walking speed comparable to that of humans (corrected walking speed (leg length/s)) [20].

**Figure 1:**
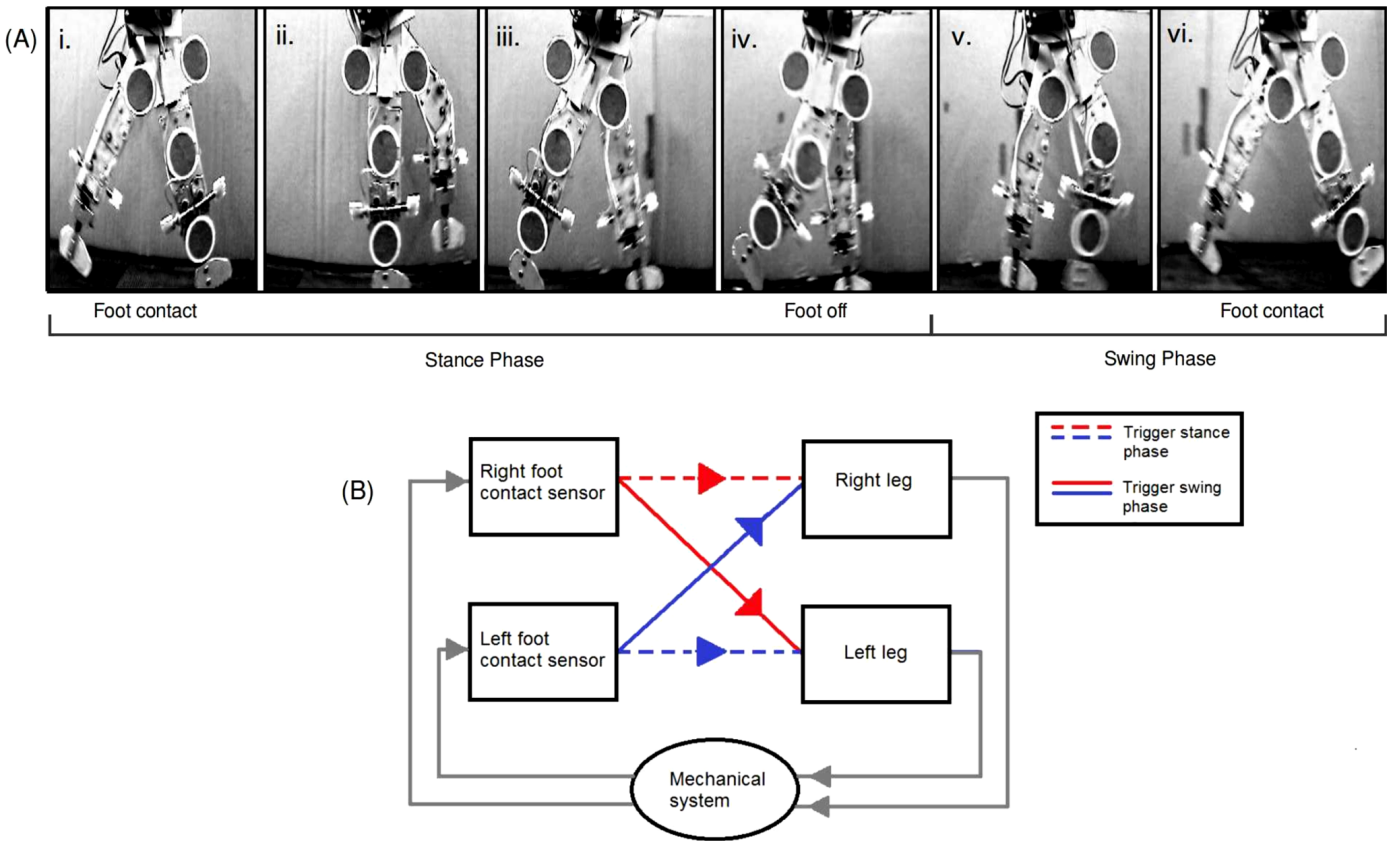
RunBot's basic operation involves phase switching of the legs triggered by contact signals from the feet. (A) Photographs of RunBot's gait cycle and (B) the system used by RunBot to generate stepping.

RunBot is driven by local reflexes without any use of position or trajectory-tracking control algorithms and without using a central pattern generator [19], [20], [22]. Phase switching of the legs is triggered by ground contact signals, when one foot contacts the ground this signal triggers motors driving hip flexion/extension and knee flexion/extension of the swing and stance legs, driving the walking forward. The reflexive locomotion controller design implemented in RunBot is based on the reflexive mechanisms observed in human gait [23], and on observation, RunBot's gait appears visually similar to human walking, Fig. 1.

Central to this paper is investigating the control between the sensor inputs of the robot and its motors. The original RunBot I attempted a biologically inspired approach where the sensor signals were translated into motor signals with the help of a neural network incorporating biologically inspired neuronal functions (see [19], [20], [22]). However the human nervous system is highly complex and has many unknown variables, in addition to controversy of how and even where the control of walking actually originates, means creating a robot with function comprised of neural networks, is highly speculative. In this paper, we go the opposite way and create an abstract controller which is based on actual human walking data instead of classical control theory. To essentially create a purely analogue closed-loop system we just require knowledge of the causal relationship between the foot contact information and the motor activation or, in the case of humans, the muscle activation (EMG). By creating a simple mechanism for generating stepping we take a black box approach to modelling the complex neural control system in humans and instead study how input signals can be translated into a functional motor output.

Our aim was to calculate transfer functions from human walking which translate sensory information into muscle activation signals by recording foot contact data and leg muscle activity (EMG) in healthy subjects as they walked on a speed controlled treadmill. To average out the periodicity in the recorded data, irregular walking patterns needed to be generated. As a treadmill can be viewed as a foreign environment for walking, which may also have an effect on the subjects walking, varying the walking speed in a random fashion should also create an environment which is more closely modelled on natural walking where speed can be changing constantly.

The unknown transfer functions which translate the contact information into muscle activation signals were identified using adaptive filtering. The filter was trained by using the heel contact information as an input (contralateral (CH) and ipsilateral (IH) to the leg muscle) and the EMG activity from the Tibialis Anterior (TA), Lateral Gastrocnemius (LG), Rectus Femoris (RF) and Biceps Femoris (BF) as the output.

The algorithm converged for the following relationships between the muscle activity and heel contact and generated stable transfer functions: 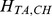, 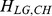, 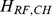, 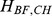, 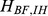. These transfer functions show that there is a causal relationship between the foot contact information and motor output. All functions could then be applied to Runbots control model to control its motors, where the Runbot was used as a biomechanical model to test the control programme.

Characteristics within the transfer functions related to flexion or extension of the hip and knee joints in humans were identified and separated to produce transfer functions for controlling RunBot's leg motors and generate stepping. The following transfer functions were applied to RunBot's hip and knee motors and were successful in producing a stable gait cycle: 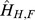, 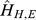, 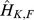 and 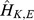, where H, F/E is hip flexion/extension and K, F/E is knee flexion/extension.

Knowledge of how sensory information from the peripheral nervous system (PNS), in humans, relates to motor actions of the muscles and limbs throughout the gait cycle has potential use in iSCI rehabilitation. Specifically if foot contact information is causally related to muscle activity, then contact information from the feet could be used as a feedback control mechanism for use with FES of leg muscles to generate walking. The idea of this approach is an entirely analogue closed-loop system to generate locomotion using simple reflexes and without central pattern generators. This has potential to provide a minimalistic control system for FES, where the cyclic sequence of joint movements is minimally imposed on the walker, which has an application for producing functional gait in individuals with iSCI without overriding any residual function which may remain. We have shown that transfer functions can be found to translate information from the feet during the gait cycle into muscle activation signals with correct timing to promote flexion and extension of the hip, knee and ankle joints (analogous to the function of RunBot's motors). The long-term aim would be the development of a device which will promote limit cycle walking, allowing the walker to adapt their gait to suit changing loading conditions dependent on terrain or the environment.

## Materials and Methods

### Ethics statement

The investigation was granted ethical approval by the University of Strathclyde ethics committee. Ten subjects, four males and six females with a mean age of 26.5 years (range 23–30 years) were recruited at the Department of Biomedical Engineering, University of Strathclyde and gave full informed written consent before taking part in the study.

### EMG and foot contact recording

**Figure 2:**
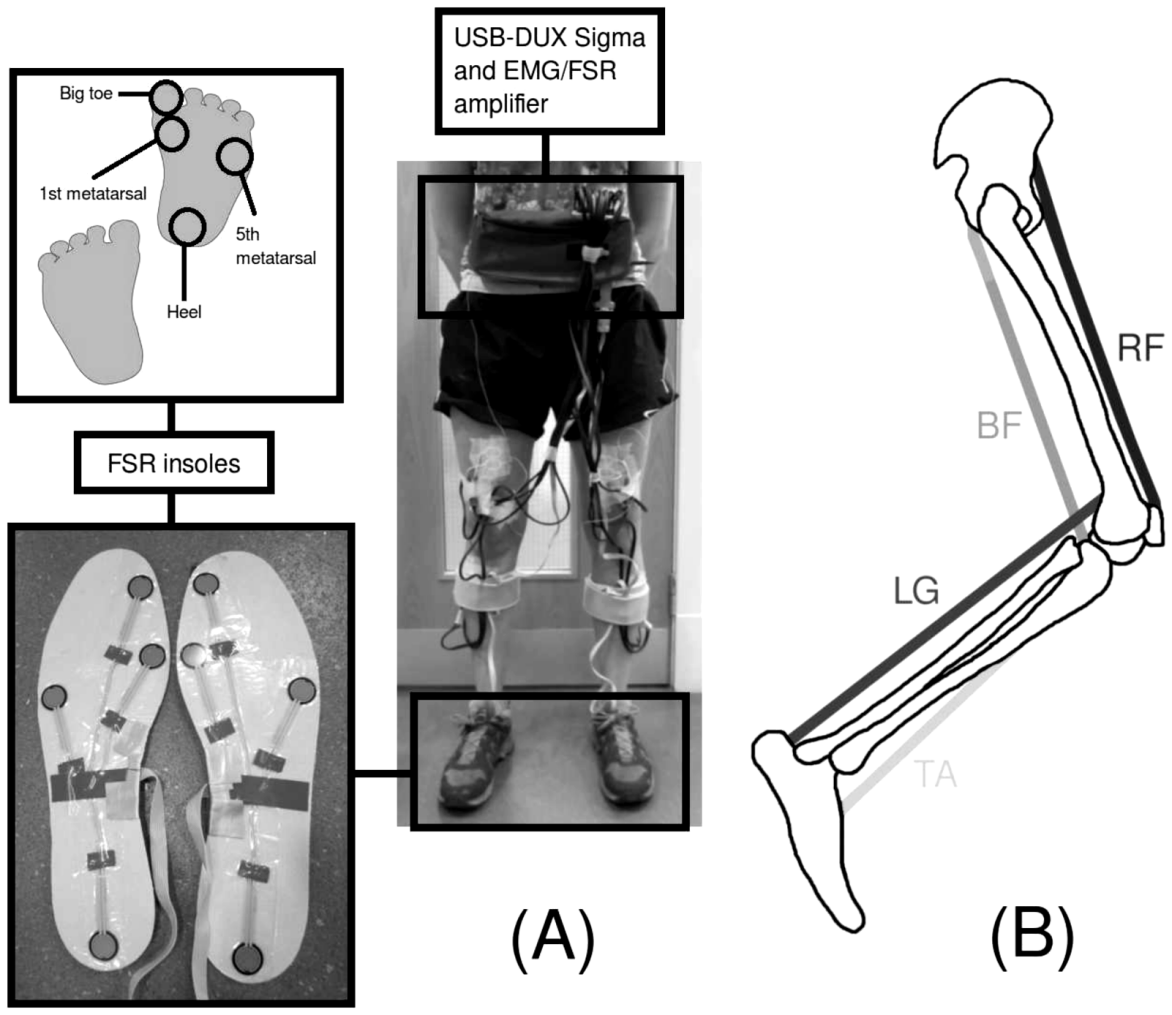
Set-up for the treadmill walking trials. (A) The USB-DUX Sigma data acquisition device and EMG/FSR amplifier are worn in a waist bag around the subject's waist. Surface EMG electrodes are used to record the muscle activity during the treadmill walking. FSR insoles are placed in the subject's shoes and measure contact signals under different areas of the feet. (B) Position of the recorded muscles on the leg. TA  =  Tibialis Anterior, RF  =  Rectus Femoris, BF  =  Biceps Femoris and LG  =  lateral Gastrocnemius.

The study involved recording muscle activity and foot contact information during treadmill walking. The muscles recorded were chosen due to their different roles in the gait cycle, two muscles located in the shank (Tibialis Anterior (TA) and Lateral Gastrocnemius (LG)) and two in the thigh (Biceps Femoris (BF) and Rectus Femoris (RF)), see Fig. 2. Bipolar surface EMG electrodes were attached parallel to the muscle fibres in the centre of the muscle belly in accordance to the recommendations of SENIAM [24]. Pre-gelled, one use surface electrodes (Blue Sensor N-10-A, Ambu, St. Ives, Cambridgeshire) were used and the skin prepared following standard procedure before application [24].

To record muscle activity and foot contact information during the treadmill walking, a purpose designed EMG/FSR amplifier was developed (PCB design files available from http://www.linux-usb-daq.co.uk/howto2/bio-sigma/). The device was required to be lightweight and compact so it could be worn by the subject during ambulation. The device has eight channels for recording surface EMG of the four leg muscles in both legs. The device also incorporates amplifier circuitry for force sensing resistors (FSRs) (Interlink Electronics, Camarillo, CA, USA) for measuring foot contact information. The FSRs are embedded in standard shoe insoles at four different positions under each of the feet for recording areas of peak pressure distribution during walking (main weight bearing areas); under the first and fifth metatarsals, big toe and heel, as described by Granat et al. [25]. Images of the experimental set-up can be seen in Fig. 2 and Video S1.

FSR insoles were constructed for each individual subject. To position the FSRs accurately under the foot aligned with the four areas of interest, FootDoc foot impression sheets (Visual Footcare Technologies, LLC, NY, USA) were used to create a template of the feet.

All sixteen of the EMG and FSR channels were recorded simultaneously with a sampling frequency of 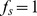 kHz using the USB-DUX Sigma data acquisition device (Incite Technology Ltd., Stirling). This device also provides a regulated 5 V power supply to the attached circuitry and electrically isolates the subject from the mains supply. The device connects via USB to a computer running Linux for data acquisition. Comedirecord (open source software available from http://www.linux-usb-daq.co.uk/software2/comedi-record) was used to record the walking data and the output saved in a MATLAB compatible ASCII file for further analysis.

### Treadmill control

A belted treadmill (Quasar Med, h/p/cosmos sports 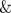 medical gmbh, Germany) was used during the study. To generate an irregular walking pattern, a control program was written in C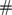 (Visual Studio 2008, Microsoft, Washington, USA and MonoDevelop 2.10, http://monodevelop.com) to produce a pseudo-random sequence of belt speed settings within a desired range. The program was based on the Coscom V3 interface protocol (available from www.coscom.org) enabling the treadmill to be controlled over a RS232 connection to a computer. The belt speed was transmitted via Ethernet and recorded alongside the EMG and foot contact data in Comedirecord.

**Figure 3:**
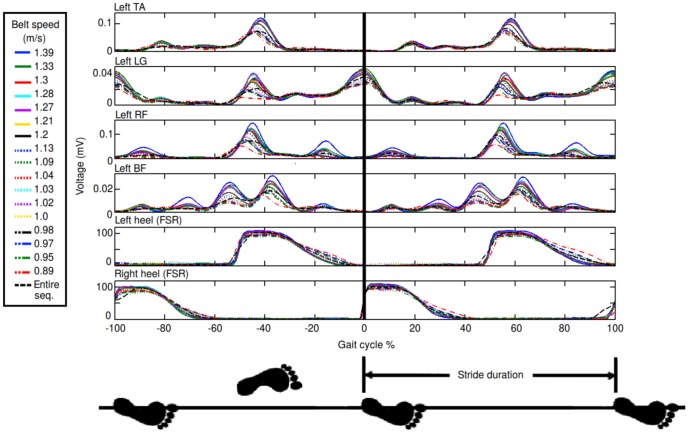
EMG and FSR foot contact data from each subject was recorded over a range of different walking speeds. The data could then be separated depending on the walking speed and compared to the activity recorded over the entire sequence (black dashed line in figure). To analyse the activity before and after heel contact, an event related average (ERA) was taken in a time period of one stride duration before and after the heel contact. The figure demonstrates the relationship between left leg smoothed and rectified EMG and heel contact information from one of the ten subjects during walking sequence 1 (25 steps per speed setting). Increasing walking speed increases the amplitude of the EMG signal, as described by [28]. TA  =  Tibialis Anterior, LG  =  Lateral Gastrocnemius, RF  =  Rectus Femoris and BF  =  Biceps Femoris.

The change in walking speed was set as small increments/decrements between 0.05 and 0.1 m/s to prevent the subject stumbling. To produce speed sequences within the natural walking speed range of each subject, measurements of gait parameters, including average walking speed and cadence, were taken prior to the treadmill walking using a wireless gait assessment device (wi-GAT) (as described in [26]). Two sequences were generated for each subject, each lasting approximately 15 minutes with a rest break given in between. The first generated sequence comprises of 20 speed settings and repeats twice. Each speed setting is programmed to run long enough to generate approximately 25 steps per speed and the complete sequence has a total range of 0.5 m/s, see Fig. 3. In comparison, the second sequence comprises of 10 speed settings, without a repeat and has a speed range of 0.39 m/s with each speed running for a longer duration to produce approximately 100 steps per speed.

Generating irregular walking using two different ratios of steps per speed setting will demonstrate which sequence produces a better average in the EMG and FSR data for calculating transfer functions. To visualise the relationship between the heel contact and EMG, an event related average (ERA) was taken in a time period of one stride duration before and after heel contact, Fig. 3. Indication that a motor neuron pool, which facilitates the specific muscle, has received suppressed or facilitatory synaptic input is given by troughs or peaks in the ERA of the rectified EMG [27]. By maintaining a walking speed range within a moderate walking speed range (0.75 to 1.75 m/s), the effect of speed on the EMG pattern can be viewed as the addition of a speed-related gain to a standard pattern [28]. Taking an ERA of the entire sequence (black dashed line in Fig. 3) provides an average of the EMG activity over all of the different walking speeds with the aim of identifying the base EMG patterns.

### Adaptive filtering

The next step is to calculate the transfer functions which translate foot contact information into muscle activation signals. These are especially useful in the creation of a human-walking model with potential applications in the development of humanoid robots with locomotion based on human walking and within rehabilitation engineering research.

Adaptive filtering was used to derive the transfer function for each of the recorded muscles and implemented using MATLAB (version 2012a, The MathWorks Inc., Natick, MA). The EMG data for each muscle in the left or right leg, 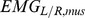 (where mus  =  TA, LG, RF or BF), was first processed using a band pass filter (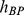) (FIR filter, 20–500 Hz) to remove artefacts, then full-wave rectified and low-pass filtered (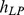) (zero-lag fourth-order IIR Butterworth filter, 6 Hz) to leave the linear envelope of the EMG.
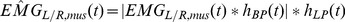
(1)


Where 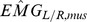 is the smoothed and rectified EMG. The EMG and FSR data recorded from each subject is provided as supplementary files, Data S1–S21.

Using the Least Mean Squares (LMS) algorithm, the output signal 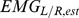 is estimated through convolution of the filter impulse response for each muscle 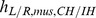, with the filter input vector 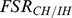, the FSR contact data from the contralateral (CH) or ipsilateral (IH) heel to the muscle.
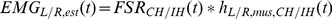
(2)


The error signal e(n) is then calculated as the difference between the desired signal 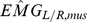 and the estimated signal 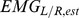.
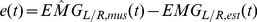
(3)


The error signal drives the optimisation algorithm which updates the filter coefficients with correction factor 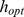 at every time instant.
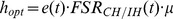
(4)


Where 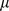 is the learning rate of the adaptive filter. The length of the response of the filter was set to the duration of two strides for each subject and the number of iterations set to 100, where the filter converges. So,
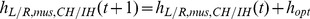
(5)


The transfer function coefficients were calculated using the adaptive filtering method for each of the four leg muscles from each of the ten subjects. A table of the final mean square error (MSE) of the filter coefficients is provided in Table S1.

**Figure 4:**
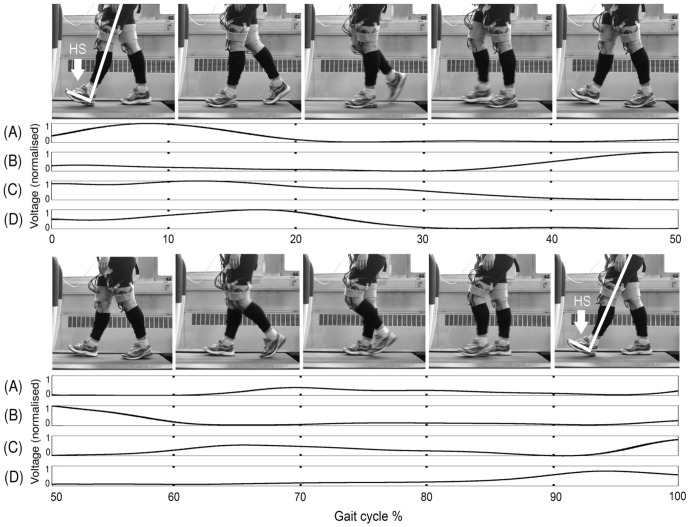
Photograph series representing one gait cycle during treadmill walking. The series of frames corresponds to one stride from heel strike of the left leg (highlighted in white in the first and last frame) to the next heel strike of the same leg. The filter output using the transfer functions for each measured muscle of the left leg corresponding to the heel strike of the ipsilateral leg, found using the adaptive filtering, are shown alongside the images of one stride duration. (A)  =  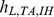, (B)  =  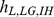, (C)  =  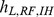 and (D)  =  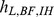, HS  =  Heel strike.

Applying the coefficients to an FIR filter produces a muscle activation signal when the filter is given an input of a typical FSR heel contact signal. A half Hanning window was convolved with the impulse response of the variable filter to select only the coefficients needed to generate a muscle activation signal one stride duration in length subsequent to the input of a heel contact signal from an FSR. An example of the filter outputs for one subject can be seen in Fig. 4 together with corresponding film frames of the subject's gait cycle (film frames taken from Video S2).

## RunBot

Before the transfer functions can be applied to the RunBot robot the standard operation and mechanical structure need to be understood in terms of how the motors can be controlled by muscle activation signals.

RunBot II is the second generation development of a biped, robotic walker which features some adaptions to the robot (RunBot I) described by Geng et al. (2006) [19], [22].

RunBot I was designed with stiff knees, which has a disadvantage of causing damage to the gearbox of the motor in the joint due to the impact of the leg on the ground at heel strike. To improve in the RunBot's knee structure and minimise damage to the joint, the motor was moved up to the thigh and three springs were positioned at the joint. This creates a balanced spring-loaded pulley using a robust bearing at the knee joint. The different springs are dominant either during flexion or extension, similar to muscles in the human leg. Using this configuration of springs, there is still a linear relationship between the motor angle and the knee angle but the knee retains an ability to flex to absorb the shock to the joint at heel strike [29]–[33]. A mechanical stop or ‘kneecap’ keeps the knee locked straight during the stance phase. This kneecap also prevents hyperextension and damage to the joint during knee extension at terminal swing.

Further development of RunBot II includes using filter functions to generate a coordinated walking behaviour rather than neuronal processing, which was the original control structure employed in RunBot I.

Filter functions and real-time processing allow fast tuning of few parameters however, like RunBot I, ground contact information is still used as the main sensory input to promote joint movement and stepping.

RunBot II has a height of 0.3 m (foot to hip joint axis) and a total weight of approximately 552 g. Motors at RunBot's hip and knee joints are driven by output signals of a reflexive control program written in C++ (running on a Linux PC) with a sampling rate of 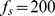 Hz through a USB-DUX D DA/AD converter board (Incite Technology Ltd., Stirling). The hips are actuated directly by DC motors (HS-625MG, Hitec RCD, USA) whereas the knees are actuated by DC motors (HS−85+MG, Hitec RCD, USA), via springs. The four (A/D) input channels of the USB-DUX measure the angles of the joints: at the left and right hip (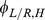) and the left and right knee (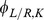). Two standard micro switches in the feet detect the ground contact: on the left (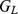) and right foot (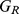). Finally, the four analogue outputs (D/A) of the USB-DUX, which have a range of 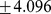, are used to drive the four motors on the hip joints 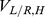 and the knee joints 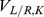 following amplification (with a gain of 2.3).

The robot has no ankle joint but features flat feet with serrated soles to increase friction with the ground and prevent slipping. RunBot is counterweighted in the sagittal plane by a weight and boom. This is connected to the robot by a joint which rotates freely in the forward direction but prevents the robot falling sideways. The boom (total length of approximately 1 m) rotates freely around a central pivot with one end attached to RunBot and the other to a counterweight. With this configuration, the robot is not being suspended or supported in an upright position but its motions are constrained to a circular path. A camera (colour board camera L79AB) is fixed to the boom arm for tracking markers positioned on RunBot's right hip, knee and ankle for gait analysis and calculation of joint kinematics.

## Reflexive Control System

RunBot's reflexive control system can be explained through description of three important events in the gait cycle:

1. Ground contact
2. Anterior extreme angle (AEA) of the contralateral hip joint
3. Passive dynamic walking phase

(1) Foot contact with the ground triggers the hip and knee of the contralateral leg to begin flexing (swing) and the ipsilateral hip and knee to begin extending (stance), Fig. 1Ai. (2) When the contralateral hip reaches the anterior extreme angle (maximum flexion position) the knee of the same leg is triggered to straighten producing leg extension at terminal swing, Fig. 1Av. (3) Once the contralateral knee has extended in preparation to contact the ground and the remaining motors have all reached the required positions, the motors are switched off. This causes the centre of gravity of the robot to shift forward of the boom leading to the foot making contact with the ground, which in turn begins the next cycle, Fig. 1Avi. In RunBot's operation, every motor switches off when the joint reaches the required position so it can be expected that during the gait cycle there may be a period where all of the joint motors are off. During this time RunBot can be termed a passive dynamic walker as the joints are not fixed in an angular position by the motors and are instead driven by the mechanism of natural dynamics acting on the structure.

In mathematical terms, the reflexive model of RunBot is a simple system involving convolution of the summed impulse trigger signals, from the leg joints and the ground contact information from both feet, with transfer functions 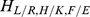 (left/right leg, hip/knee joint, flexor/extensor). Ground contact switches trigger an impulse signal from the left (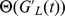) and right foot (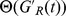), where 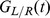 is 1 when the foot contacts the ground and 0 with no contact so 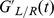 is the derivative impulse, and are the main inputs to the controller. There is also a local joint control feature for preventing the over-flexion or extension of the joints by calculation of the angle from the motor voltage. The total motor output of each of the four leg motors are defined by 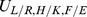 and drive the walking behaviour, Eqn. 6a, 6b, 6c and 6d.

Shown are the general equations for both legs, with ‘I’ defining the ipsilateral leg and ‘C’ the contralateral leg:
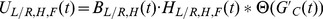
(6a)

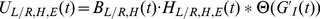
(6b)

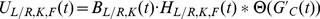
(6c)

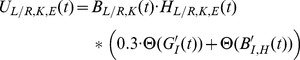
(6d)


Where 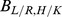 is a parameter preventing the joints flexing or extending beyond an extreme angle threshold (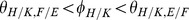) by limiting the motor voltages to prevent mechanical damage. 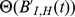 is used as an impulse trigger signal to trigger knee extension of the ipsilateral leg at terminal swing when the anterior extreme angle (AEA) of the hip is detected (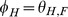), Eqn. 6d. The values used for the extreme joint angles can be found in the Table S2. These values were hand-tuned as described in [19], [22].

The final outputs 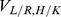 to the USB-DUX are found by multiplying 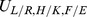 with predefined gain coefficients, where 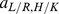 is the gain of the motor amplifier (the gain values of the hip and knee motors are provided in the Table S3). As with the extreme joint angle values, the gain of the motor amplifier was chosen intuitively in accordance with the method used by [19], [22].
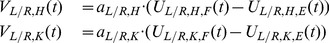
(7)


### Generating RunBot's walking using human-derived transfer functions

After having established the transfer functions using the human treadmill walking data and adaptive filtering (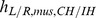, Eqn. 5) which connect the heel contact and muscle activity in the legs, we next need to translate them over to the RunBot. As the hip joint and knee joint controls are separate in RunBot, the features of the human muscle transfer functions needed to be separated according to specific function (e.g hip flexion, hip extension, knee flexion and knee extension). It is also necessary to define the triggers for the transfer function and resample and normalise the functions in accordance with RunBot's control mechanism.

As we have discussed, RunBot has push switches in its feet which generate impulses on contact with the ground (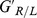) to trigger motor switching in the knee and hip joints. However during the treadmill walking study, foot contact information was recorded using FSRs positioned under the feet which produce an increasing voltage curve when pressure is applied. To compensate for the difference in foot contact measurement between the two systems, and enable the human derived muscle transfer functions to be applied to the RunBot, the transfer functions calculated using the FSR data 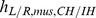 were convolved with the mean FSR heel contact signal one stride duration in length for each subject, 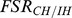.
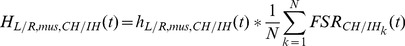
(8)


Where N is the total number of strides recorded during the treadmill walking.

The effect is that the response of the filter to an impulse becomes equivalent to applying an input of a typical heel contact FSR signal measured during gait and RunBot can still use its original foot contact impulse trigger signal (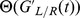).

We are looking to define the functions 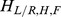, 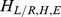, 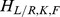 and 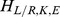 which relate to those presented in Eqn. 6a, 6b, 6c and 6d.

**Figure 5:**
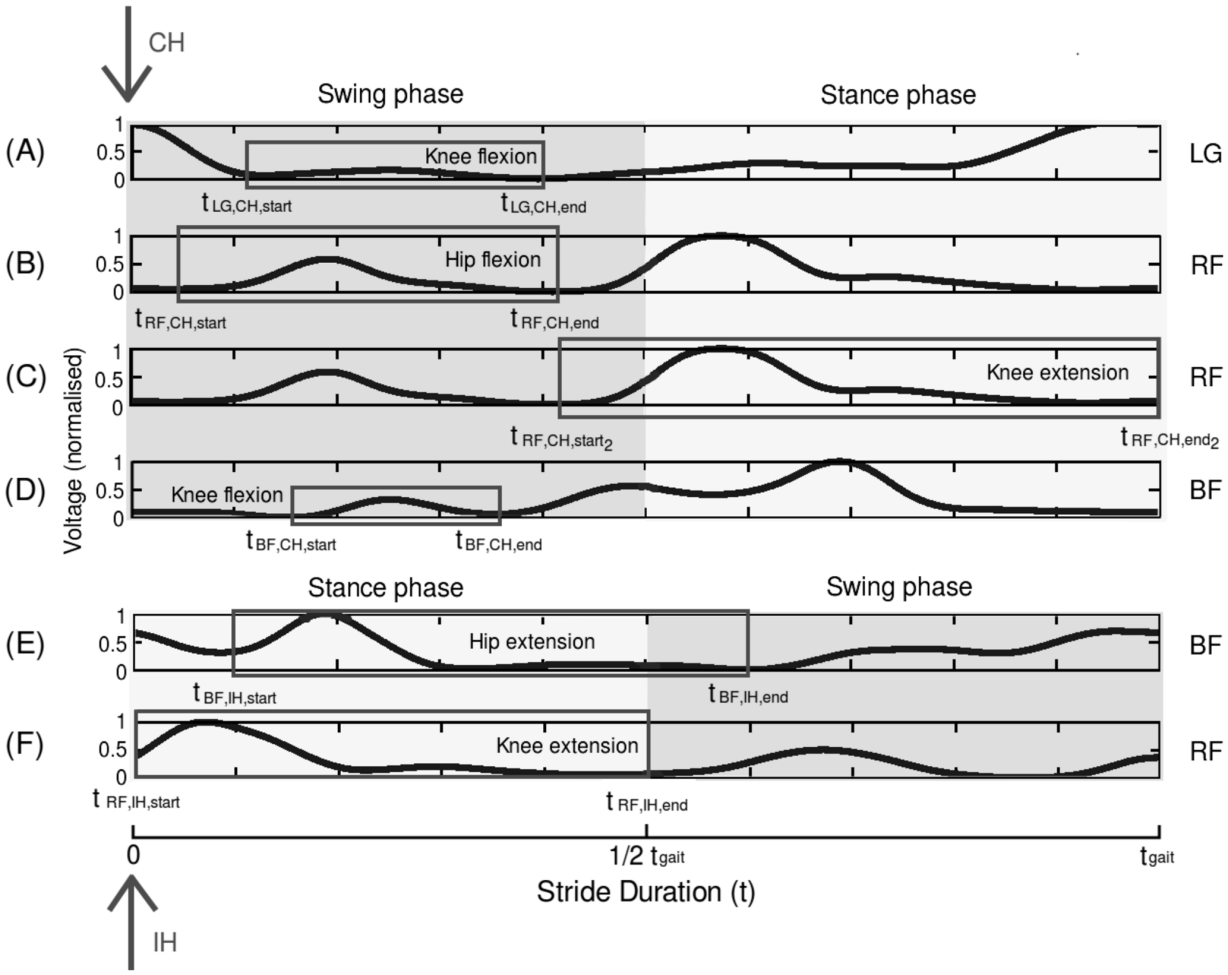
Identifying features of the transfer function coefficients which correspond to muscle activity promoting knee and hip flexion/extension in human walking. The transfer functions from adaptive filtering heel contact data from the contralateral and ipsilateral foot to the specific leg muscle ((A) 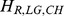, (B+C) 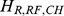, (D) 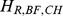, (E) 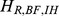 and (F) 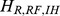) were used to identify the required features. These coefficients were then used in an FIR filter to control motors in RunBot's hip and knee using the sensory input of the contralateral or ipsilateral heel contact.

By examining the muscle activity relating to one stride duration, we can analyse the peaks and troughs in the data and correspond this to knowledge of the muscle actions during the gait cycle and analysis of video footage of each subject during the treadmill walking (see Fig. 4 and 5 and Video S2, which provides an example of a subject walking on the treadmill) to identify the information in the signal which could be used to control RunBot's hip and knee motors. The action of each muscle on the hip and knee joint motion is summarised in Table 1. The trigger for each of these events is either the contralateral heel contact (CH) or the ipsilateral heel contact (IH).

**Table 1:** Relating the muscle transfer function to RunBot's motor control.

Transfer Function	RunBot Motor Control
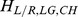	Knee flexion during swing (CH).
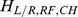	Knee extension at terminal swing and during the stance phase (CH).
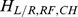	Hip flexion during swing (CH).
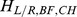	Knee flexion during the swing phase (CH).
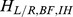	Hip extension during the stance phase (IH).

As the ankle joint in the current RunBot II is rigid, the recorded TA activity was not considered relevant as the muscle only has action on the ankle joint in humans. The other three muscles are all bi-functional muscles with action on either the hip or knee joint or both (Table 1).

The peaks and troughs visible in the EMG transfer functions, Fig. 5, relate to activation and suppression of the motor neurons. To separate the activity into the transfer functions relating to the joint movement, the data located between the minimum value of the trough preceding the peak to the subsequent trough minimum value was selected.

Using the aforementioned information we can now discuss how each muscle transfer function is transformed into transfer functions for controlling RunBot's motors.

#### Rectus Femoris (RF)

RF is responsible for hip flexion (in the swing phase) and knee extension (in the late swing phase and the stance phase). Two separate peaks of activity can be observed in the RF transfer function 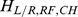 (identified by a box in Fig. 5B, C and F). As the first peak is during the swing phase, the activity corresponds to hip flexion and the second peak, which coincides with terminal swing, is identified as activity related to knee extension.
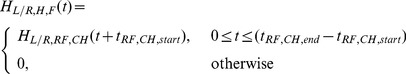
(9)

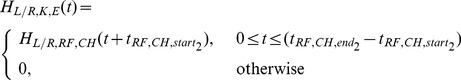
(10)


Where 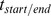 is the identifiable trough before and after the peak in the data associated with the hip/knee flexion/extension and 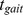 is the total duration of the gait cycle (i.e 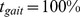). 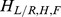 can then be substituted directly into Eqn. 6a.

Our aim was to relate muscle activity to foot contact and use this to trigger muscle activation signals with the purpose to attempt to represent the underlying muscle activation dynamics [12]. This theory can be realised when comparing the timing of muscle activity with heel contact information. The only exception where the muscle activity does not follow heel contact is the knee extension at terminal swing which occurs approximately between 40 and 50% of stance before ipsilateral heel contact at 50%, Fig. 5C. An alternative, analogous to human walking, involves angular sensory information from the hip or knee joint to trigger the knee extension. This corresponds to the reflexive neuronal control model currently implemented in RunBot II under its normal operation, see Eqn. 6d. We used the Anterior Extreme Angle (AEA) of the hip joint as the trigger signal of 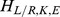, Eqn. 10, instead of foot contact. When the hip flexion angle reaches a threshold, the knee motor extends the leg to prepare for foot contact with the ground. For RunBot's reflexive controller we replace Eqn. 6d (for the knee extensor) with:
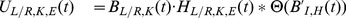
(11)


Where, as previously, 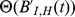 is the impulse trigger signal when the ipsilateral hip reaches the AEA.

#### Biceps Femoris (BF)

BF is responsible for hip extension (in the stance phase) and knee flexion (in the swing phase), two motions in different phases of the gait cycle. By taking the transfer functions derived from the BF EMG activity and ground contact information from both feet (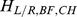 and 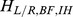) we can identify the peak activity following the contralateral heel contact trigger signal as the knee flexion transfer function (highlighted by a box in Fig. 5D) and the hip extension transfer function (Fig. 5E) following ipsilateral heel contact.
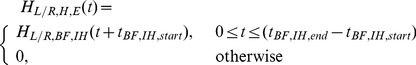
(12)



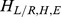
 can be substituted in Eqn. 6b and used for hip extension in RunBot's reflexive control system.
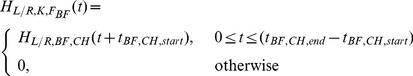
(13)


#### Lateral Gastrocnemius (LG)

The LG transfer function is responsible for ankle dorsiflexion in the stance phase (body weight supporting in mid-stance phase and heel off motion in terminal stance phase, toe off in pre-swing phase) and knee flexion in pre-swing and initial swing phase. The transfer function relating to contralateral heel contact (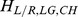) has a peak coinciding with knee flexion following toe off and this feature can be used with RunBot to generate knee flexion triggered by contralateral heel contact (Feature of interest highlighted in Fig. 5A).
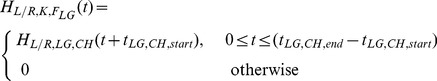
(14)


In the case of two of our recorded muscles being responsible for the same action (e.g LG and BF in knee flexion) the sum of the two transfer function coefficients is taken. The sum can then be substituted in Eqn. 6c for knee flexion in RunBot's control system:
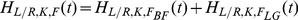
(15)


The start point for the transfer functions next needs to be defined. It is important to note that the data is cyclic and thus a start and endpoint of the function needs to be determined.

As we have already discussed, the delay between contralateral heel contact and muscle activity related to knee extension at terminal swing (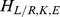, Eqn. 10) is too large for the heel contact to be deemed a suitable trigger for this action. Using hip AEA as the trigger means that the transfer function start point is taken as the time of the trough minimum (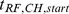) which precedes the peak of activity related to knee extension. In this way the filter is triggered immediately when the hip AEA is reached.

The springs used in RunBot's knees produce a latency period due to a delay between the motor turning and the springs reacting which can be viewed as equivalent to the delay between heel strike and toe off of the contralateral foot observed in normal human walking during the double support phase (first 10%) of the gait cycle, see Fig. 4.

Unlike the knee, the hip joint motor in RunBot is directly controlled by the motor so there is no spring latency period. The delay in motor activation between heel strike and contralateral toe off in the transfer functions, summed with RunBot's spring latency period, produces an extended delay causing knee motion uncoordinated to the hip. For this reason, the delay between the trigger and the onset of knee flexor activity was subtracted from the knee flexion transfer functions. As the hip joint motor is not controlled by springs the delay between trigger and muscle activation for hip joint control was not removed.

### Curve fitting

The final stage in data processing before applying the transfer functions to RunBot is curve fitting to remove spurious artefacts in the EMG as the assumption is made that a muscle activation signal should be a smooth increase and decrease in voltage with contraction.

The muscle twitch response of muscle has a characteristic shape which closely matches the impulse-response time curve of a damped, linear second-order differential system and models the net result of coupling between the excitation and contraction of the muscle [34]. The second-order model behaves essentially like a low-pass filter producing a delay between the neural excitation and the active state of the muscle [35], [36].

To this purpose we have used the impulse response of a critically damped system to curve fit the muscle excitation of the desired features of the muscle transfer functions using the least mean squares (LMS) algorithm and model optimisation in MATLAB. The resulting transfer functions are (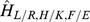) which can be applied at RunBot's hip and knee motors (H/K) for flexion or extension (F/E).
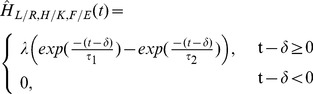
(16)


Where 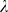 is the amplitude fitted variable. 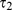 and 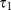 are equivalent to the rise and fall time respectively and 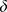 is the delay constant from the trigger signal to the onset of muscle contraction.

Only the positive values of the curve fitted transfer function were taken and normalised to an amplitude range between 0 and 1 V. This enabled the motor voltage to be easily adjusted according to the observed gait pattern stability using Eqn. 7.

The transfer functions correspond to one stride duration defined as from foot contact to the next foot contact of the same leg. The mechanical system mainly dictates how the transfer functions need to be resampled for the RunBot. The transfer functions for the hip and knee motors were sampled at the frequency of the control program (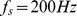 or one second) for one stride duration and the knee motor transfer functions were subsequently halved to a duration of 500 ms, for one step. The results of the curve fitting are provided in Table 2 and 3.

**Table 2:** Results of the curve fitting for hip flexion/extension.

	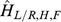			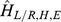		
Set	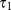 (ms)	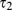 (ms)	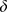 (ms)	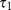 (ms)	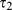 (ms)	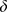 (ms)
1A	76.97	76.96	75	73.22	73.22	75
1B	86.91	86.91	80	128.24	81.73	85
2A	88.31	88.31	30	72.47	72.47	80
2B	83.80	83.80	60	71.82	71.82	80
3A	78.53	78.52	90	72.31	72.31	100
3B	93.71	93.71	80	133.76	133.76	80
4A	91.96	91.96	125	95.88	34.27	80
4B	77.52	77.52	70	100.46	100.46	50
5A	113.28	113.28	5	91.43	91.43	15
5B	76.18	76.18	75	74.65	74.65	100
6A	93.08	93.08	110	112.16	112.16	100
6B	78.41	78.41	120	110.89	110.89	100

Values are provided for 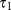, 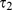 and 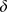 which can be substituted into Eqn. 16.

**Table 3:** Results of the curve fitting for knee flexion/extension.

	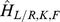			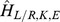		
Set	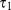 (ms)	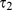 (ms)	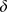 (ms)	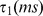	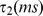	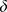 (ms)
1A	83.11	83.11	115	103.84	103.84	425
1B	105.02	105.02	115	134.63	134.59	440
2A	94.47	94.47	110	107.71	107.71	430
2B	76.58	76.58	120	139.47	139.47	435
3A	69.09	69.09	140	77.13	77.12	460
3B	113.76	113.76	125	94.56	94.56	495
4A	108.96	108.96	90	136.61	136.54	450
4B	82.99	82.85	105	150.33	150.33	415
5A	95.95	95.95	110	131.17	131.17	410
5B	82.99	82.85	105	148.99	148.99	420
6A	78.37	78.37	150	151.75	151.75	425
6B	97.24	97.22	130	123.34	123.34	495

Values are provided for 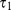, 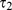 and 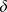 which can be substituted into Eqn. 16. As RunBot has a knee structure controlled by a motor and springs, the 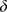 values for the knee joint transfer functions which correspond to the delay between the trigger and 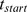, were set to zero.

In conclusion we have defined the transfer functions 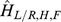, 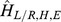, 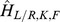 and 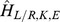 which can be substituted into the equations used within RunBot's reflexive control system, Eqn. 6a, 6b, 6c and 11. The other parameters within the control system measuring joint extreme flexion/extension angles (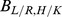) remain unchanged in order to prevent damage to RunBot's mechanical structure. The angles utilised to signal AEA of the hip joint (which promote knee extension of the ipsilateral leg at terminal swing) were also maintained, see Table S2.

The final equations for both legs which define RunBot's control system are as follows:
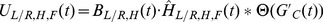
(17a)

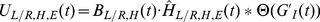
(17b)

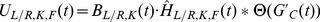
(17c)

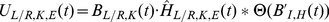
(17d)


## Results

In this section we will first discuss the results of calculating the transfer functions from the EMG and foot contact data followed by the results of applying the transfer functions to RunBot's reflexive control system.

### Transfer functions

Recall, that the final functions after curve fitting were defined as 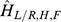, 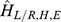, 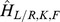 and 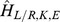, and were applied to RunBot using Eqn. 17a, 17b, 17c and 17d. To analyse the transfer functions in generating walking with RunBot, 12 different transfer function sets were applied and tested using the reflexive model to identify the robustness of the employed methodology in defining the transfer functions and whether the two different treadmill walking trials have an effect on the functions, see Table 4. Identical transfer functions were applied to the right and left legs as the assumption was made that the activity in both legs was the same.

**Table 4:** Sets of transfer functions applied to RunBot's control system.

Set No.	Description
1A/B	Mean average of all subjects.
2A/B	Mean average of male subjects only.
3A/B	Mean average of female subjects only.
4A/B	Each transfer function is from the subject who had the
	minimum final MSE value from the adaptive filtering.
5A/B	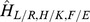 from a single male subject (subject C).
6A/B	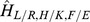 from a single female subject (subject H).

Different sets of transfer functions were applied to RunBot's control system to establish whether a stable gait pattern can be produced by combining transfer functions from the range of subjects or by just using functions from individual subjects. We also wanted to examine whether there is a difference between the two treadmill walking trials, where A  =  sequence 1 (25 steps per speed) and B  =  sequence 2 (100 steps per speed). For example, set 5A is using transfer functions from a single male subject (subject C) from treadmill sequence 1. Videos S3 to S14 show RunBot walking using each of these sets of transfer functions.

### RunBot performance

The next stages were to apply the different transfer functions sets to the defined reflexive control system in RunBot, and to analyse the resultant gait.

**Figure 6:**
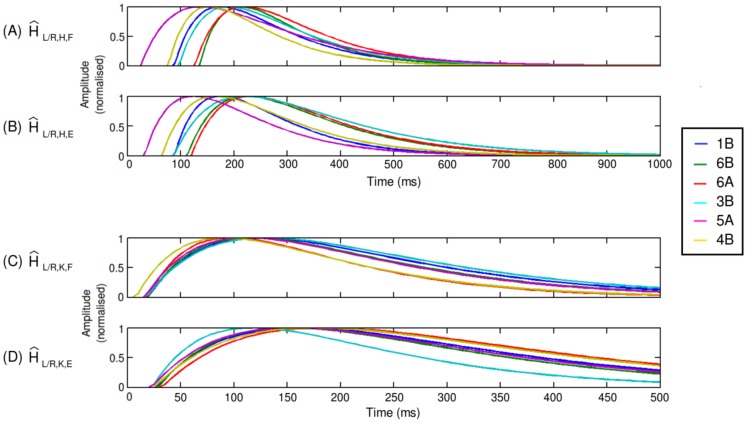
Plots of the different transfer functions tested with RunBot which produced a stable gait. The number of samples for the hip motors was set to 200 (1000 ms). This is the same frequency used during the normal operation of RunBot II. Knee flexion/extension was set to 100 samples or 500 ms. (A) Represents the transfer function coefficients from the curve fitting for the hip flexion. Hip flexion of the leg is triggered by the contralateral heel strike. (B) Hip extension is triggered by the ipsilateral heel strike. (C) Knee flexion of the leg is triggered by the contralateral heel strike and knee extension (D) is triggered by the anterior extreme angle (AEA) of the hip to drive knee extension at terminal swing.

Stable walking was defined as a controlled gait cycle with no stumbles or falls for more than 10 rotations of the circular path. Transfer function sets: 1A, 5B, 3A, 2A, 2B and 4A caused RunBot to stumble and fall while the remainder: 1B, 5A, 6A, 6B, 3B and 4B produced a stable gait pattern (See Videos S3 to S14). Comparison plots of the calculated transfer functions which worked with RunBot can be seen in Fig. 6. On comparing the function characteristics (Table 5) an obvious difference was found in the hip extensor transfer functions that produced stable gait compared to those sets which did not. The sets which featured a longer 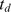 (where 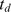 is the time period from 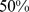 of the peak amplitude on the rise to 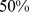 of the amplitude on the fall) in the hip extensor function were more likely to produce a stable gait. From this information we can determine that if 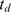 is too short, the stance hip cannot extend backwards to the desired angle which will cause the foot of the swing leg to scuff the ground and cause a stumble. There was no consistent difference between different knee transfer functions between the sets that worked and those which did not.

**Table 5:** Comparison of function characteristics.

		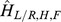		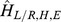	
Gait	Set	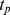 (ms)	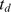 (ms)	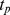 (ms)	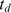 (ms)
Stable	1B	175	215	190	255
	3B	190	230	220	330
	4B	155	190	165	245
	5A	140	275	125	225
	6A	220	230	230	275
	6B	215	195	225	270
Unstable	1A	160	190	160	180
	2A	135	215	130	175
	2B	155	205	165	175
	3A	280	195	185	180
	4A	225	225	130	145
	5B	155	185	185	185

The duration (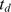) and peak time (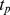) of the hip transfer functions were compared to determine the influence on whether RunBot's gait is stable or unstable. 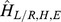 needs to have a longer duration than 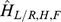 to produce a stable gait pattern.

**Figure 7:**
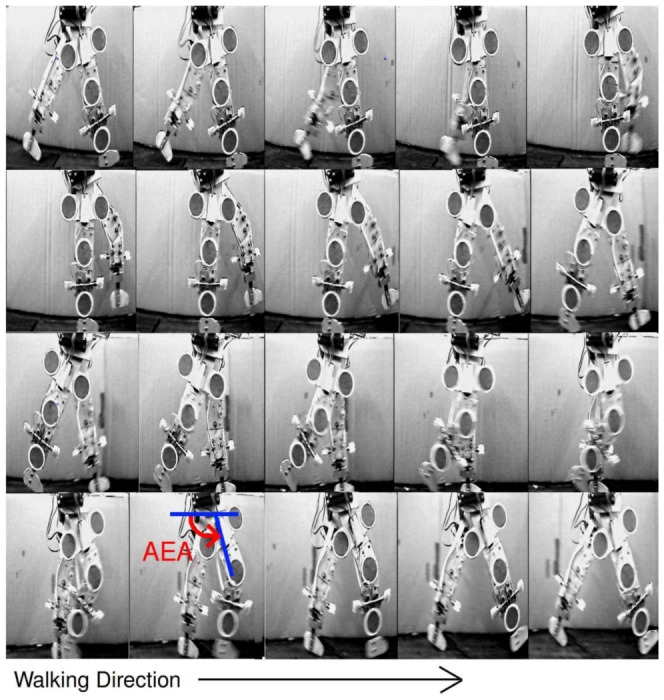
Photographs of one RunBot stride duration. The series of frames corresponds to one stride recorded after applying transfer functions found from the human study. The time interval between each adjacent frame is 60 ms. Markers were attached to RunBot's right leg for video tacking of the joints for calculation of kinematic data. Heel contact triggers the stance phase of ipsilateral leg and the swing phase of the contralateral leg. Leg extension during terminal swing is triggered by the threshold value for the hip anterior extreme angle (AEA) being reached during hip flexion.

To analyse RunBot's gait using the different transfer function coefficients, RunBot's limbs were video tracked as it walked in a circular path, see Fig. 7. A step is initiated when the stance leg foot makes contact with the ground. The swing leg hip then flexes forward and the knee flexes, lifting the foot off the ground. Once the hip reaches the anterior extreme angle (AEA) the knee is triggered into extension until the foot of the swing leg makes contact with the ground. This then triggers the contralateral leg motors and so on.

**Figure 8:**
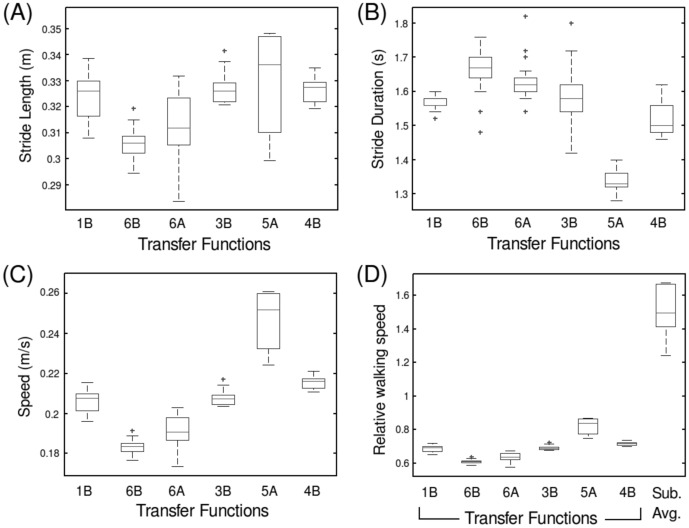
Box plots comparing RunBot's stride length (A), stride duration (B) and walking speed (C). Using the transfer function sets which produced stable walking (n = 10). A box plot comparing the relative walking speed of RunBot using each of the transfer function sets compared to the average relative walking speed of the human test subjects is also provided (D). Relative walking speed of leg-length/s is calculated as the scaled walking speed to leg length where RunBot's leg length is 0.3 m.

During joint tracking, measurements were taken of average walking speed, stride length and the knee joint angle. Fig. 8 describes how the walking speed performance of RunBot responds to the different transfer function sets. The speed result was calculated as the circumference of the cycle path 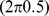 divided by the time for completing one circuit. The stride duration was calculated as the time for RunBot to complete a rotation of the circular path divided by the number of strides recorded.

**Figure 9:**
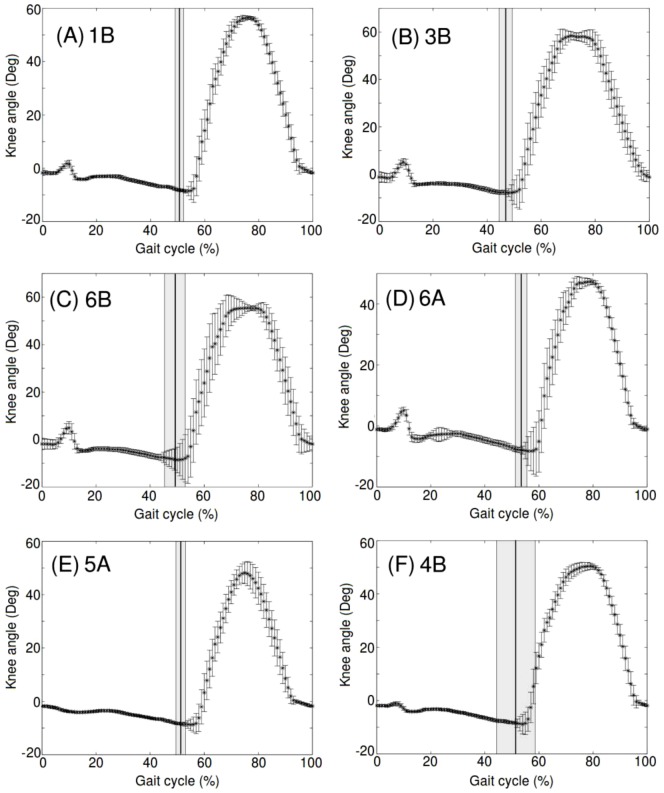
Comparison of knee flexion/extension angle of RunBot using transfer function sets which produced a stable gait. The time is normalised to percent of stride, the mean and standard deviation was calculated from the number of strides recorded from the video tracking. The mean percent of stride when the contralateral heel strike was recorded is also shown as a line with the standard deviation highlighted.

Flexion/extension angle of the knee was calculated to compare the different transfer function's effect on RunBot, Fig. 9. RunBot II has a knee structure which involves springs to mimic the muscle properties around the human knee joint due to muscle having linear, spring-like properties. Due to the knee mechanics being analogous to humans we can study the knee angle during the gait cycle to analyse the difference in transfer function from the different transfer function sets which produced a stable gait. Comparing the averages and female and male individual subject transfer function sets, the timing for stance and swing is very similar; the main difference being in the small peak evident during stance when some of the transfer function sets are applied to RunBot. This is due to the knee bending following heel strike because the hip has continued to flex after heel contact and so has pulled the knee into flexion before extension begins. In comparison to human knee flexion/extension angle during gait the plots are very similar. The major difference is that in humans there is a small flexion peak present during stance before swing begins which is more significant than the small duration peak in RunBot's knee motion. In humans this peak is due to the knee bending following heel strike as the body weight is accepted and transferred onto the leg as the swing phase of the contralateral leg begins. It also acts to absorb the impact of the heel strike by extending the contraction period of the quadriceps muscles.

Another point of interest is in the 3B and 6B knee angle curves, Fig. 9B and C. The flat peak during knee flexion in the swing phase is due to the knee flexing to its maximal angle and remaining in this state before the hip reaches the AEA (which triggers knee extension). This is in contrast to the other data sets applied to RunBot and the human knee angle example which demonstrate a more fluid movement from knee flexion to knee extension.

**Figure 10:**
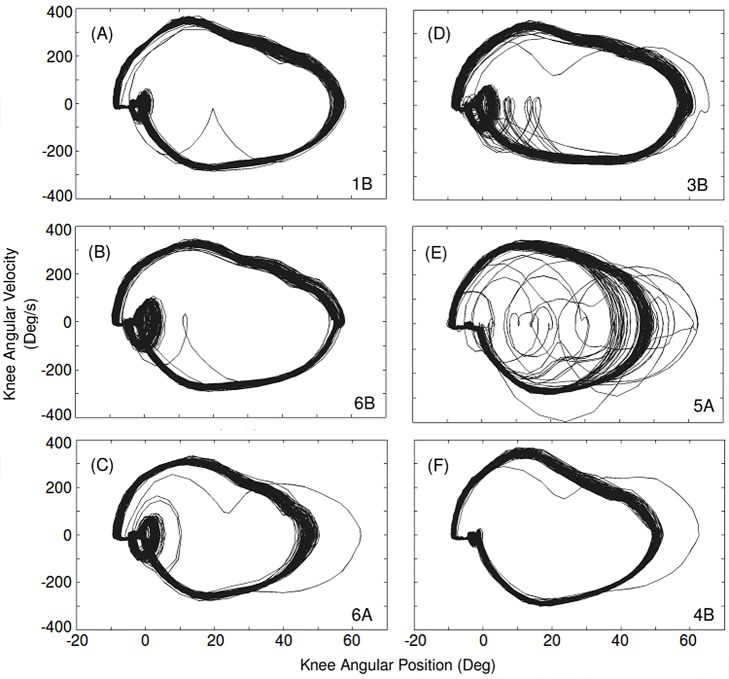
Phase plots of knee angular velocity versus angular position. Knee angle was calculated from markers positioned on the knee joint and video camera tracking over ten complete rotations of the circular path. The plots show the limit cycles in the phase plane and demonstrate the robustness of the reflexive control system, as even when there is a disturbance to the gait cycle there is a rapid convergence to the limit cycle in only a few steps.

To analyse the dynamic stability of RunBot using the different transfer function sets, phase plots of knee angular velocity versus the angular position were used, as the movement pattern is cyclic and we want to see how the performance varies over time, Fig. 10. Although the gait stability is affected by using the different transfer function sets, we can see that overall the reflexive control system produces stable limit cycles. This demonstrates that even when there is a disturbance to the gait pattern originating from an unevenness of the ground surface, there is a quick return to the steady-state behaviour. Fig. 10E is transfer function set 5A, this set produced the fastest walking speed with RunBot but the phase plot demonstrates that the limit-cycles are significantly more affected by perturbations than the other sets and so appears less stable.

Finally, we are now able to identify characteristics of the transfer functions which worked to produce stable walking in RunBot and explain why other sets did not:

- Compared to the knee transfer functions, the differences in hip transfer functions have a more significant effect on the walking performance as the hip transfer functions are used to drive the hip motor directly.
- The time delay between the trigger and the hip flexion and between the trigger and the hip extension of the contralateral leg should be very similar or the hip flexion should be longer to produce a stable gait.
- The duration 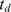 of hip extension (from 50% of the peak amplitude on the rise to 50% on the fall) is significant in establishing a stable gait pattern because the stance leg needs enough torque to support the body weight and extend the leg backward while the swing leg flexes forward.
- The duration of hip extension should be longer than hip flexion to produce a stable gait.

## Discussion

The development of a reflexive control system based on filter functions calculated from human walking data aimed to demonstrate that using sensory feedback can be a successful method to generate stable and coordinated limit cycle stepping in a robotic walker. We have shown that there is a direct causal relationship between foot contact information and muscle activity during biped walking. This causal relationship allowed us to calculate filter functions using established filtering techniques, which reproduce the activations of the relevant muscles after foot contact. Our reflexive controller exploits the natural dynamics of the robot for motion generation without the requirement of central pattern generators, trajectory planning or tracking control.

As this is an analogue linear system using foot contact as the main source of feedback, the system has high reliability where the output is dependent on there being an input. This means that the system can never enter any unknown or unpredictable state as it is not a finite state machine and uses no threshold on the input to determine the output state. If there was a loss of feedback information relating to foot contact, there would be no output from the system. We never experienced a loss of foot contact feedback in using this system with the RunBot robot or during the data collection with human participants, although a failure of the foot contact sensors could occur with potential dangerous consequences for the biped locomotion. The FSRs used in the study have a typical operation beyond 1,000,000 actuations [37], making them suitable for use in the detection of foot contact, for which they have been used previously [25], [26], [38]. Future development of the system to improve robustness and fault tolerance could involve integration of internal forward models with efference copy. As walking is a cyclic and repetitive process, the system could calculate a prediction (forward model) of the output and if the actual and predicted outputs differed the system would halt, bringing the walker to a standing stop.

Foot contact is commonly recorded for use in gait analysis as a method of determining spatial and temporal parameters such as stride length, cadence and predicting the onset and timing of gait cycle events. This information can be used alongside EMG data for analysis of muscle function to classify normal and identify pathological gait [39]. For this purpose, the generalised muscle activity patterns in relation to a normalised gait phase (0 to 100%) have been long documented [40]. In addition, different strategies for generating control based on muscle activity and foot contact information have been studied by others for use in research on human motor control and in rehabilitation engineering. These include simulated systems based on human data and control derived directly from biosignals, an area of research commonly known as brain-computer interfaces (BCI), for review [41]. However, to the author's knowledge, the transfer functions which directly relate foot contact and muscle activity within humans, either averaged from a population or from an individual, have not been calculated to create a minimalistic, linear, analogue control system for applications in gait control.

The relationship between muscle activity and walking speed is of interest as it influences how foot contact information could be used as a trigger for muscle activation. It has been documented that the stance phase of gait decreases as speed increases [42]. And it has been shown that the timing of certain gait phases occurs earlier in relative stride time as speed increases, particularly in the TA, LG, and RF muscles. The EMG patterns also tend to become more consistent with an increase in walking speed, with slow speeds causing an EMG pattern dependent on the muscle characteristics and motion of the specific individual [43]. However, changes to the gait phase timing of muscle activity during ramp acceleration and deceleration is less well understood with the majority of studies focusing on EMG analysis at constant walking speeds. Our results demonstrate that the first treadmill sequence (25 steps per speed) may have been too variable, producing an average of the muscle activity with gait timing influenced detrimentally by the periods of acceleration and deceleration between the constant speeds. The second sequence provided a higher number of complete gait cycles at every steady-state speed which produced an average which was less skewed by the periods of acceleration and deceleration. Another reason for the variation in results could be due to the range of treadmill belt speeds used in the second sequence. This range was 0.11 m/s smaller than the first which may have produced a stride average which was more compatible with the RunBot's mechanical structure. As RunBot's design is not a scale representation of human leg length and mass ratios, the faster walking speeds used in the first sequence may have produced stance to swing ratios which were incompatible with RunBot's design and construction.

Comparing the walking speeds achieved using each of the different transfer function sets, set 5A produced the fastest average speed which was from a single male subject. However when scaled to leg length (Fig. 8), RunBot's walking speed is approximately half of what the human subjects achieved walking at a constant average speed. This could be attributed to reduced energy efficiency in the robot's mechanical design by lack of an actuated ankle joint which would provide the addition of a push off force from the foot at pre-swing. Currently the knee motor has to lift the weight of the lower leg without major contribution from a ground reaction force. It would be interesting to examine whether the addition of an actuated ankle joint in RunBot, controlled using human muscle transfer functions as in the hip and knee motors, increased the relative walking speed of the robot significantly.

In human walking, several studies have indicated that ground reaction forces influence the locomotor activity of the leg [44]–[46] and the action of plantar pressure signals from the foot sole have been implicated in the reflex regulation of locomotion [47]–[49]. Research involving spinalised and decerebrated animals has suggested that afferents from the foot sole interact with the neuronal circuits involved in stepping. Sensory afferents in the sole of the foot signal spinal interneuronal circuits which can delay or suppress the initiation of swing, encouraging the stance phase as well as contribute to the correct placement of the foot during stepping [48], [50]–[52]. Load receptors can also act to signal unloading of the limb following heel strike of the contralateral leg and contribute to the termination of stance [53]. There is a significant amount of afferent activity originating from the skin of the foot after ground contact [54], which suggests there is potential that this information could be used to reinforce the ongoing muscle activations during stance. In addition, research studying electrical stimulation of nerves that supply the skin of the foot suggests that strong reflex activations in various leg muscles can be triggered during human gait [55].

It can be considered that sensory feedback from the foot sole may be of major significance in the control of human walking. Our reflexive controller has used heel contact as a sensory input trigger to activate muscles relating to flexion/extension of the hip or knee joints. The only exception to the rule was employing the hip anterior extreme angle (AEA) to determine the moment for knee extension at terminal swing because for this event there is no causal relationship between heel contact and muscle activity. A stable transition from swing to stance is dependent on the swing leg becoming sufficiently protracted before ground contact. For this reason position of the hip is a suitable candidate for producing an afferent signal regulating swing-to-stance transition [56]. A direct connection between joint angle and motor output is inspired by monosynaptic reflexes found in different animals [57] and also in humans [23]. When the limb of an animal reaches an extreme position, stretch receptors signal the controller to reset the phase of the limbs [21]. The role of hip position in regulating the stance-to- swing transition has been well documented within an animal model [58], [59] and in human infant stepping [60], [61]. Hip angle contribution to swing-to-stance transition during the swing phase of walking is indicated as the position of the hip closely reflects the forward motion of the leg. Studies involving decerebrate cats found that assisting flexion movements of the hip joint shortened the burst duration within the Iliopsoas hip flexor muscles and promoted early onset of activity in the Medial Gastrocnemius producing ankle extension. This is significant as burst activity in ankle and knee extensors occurs at the swing-to-stance transition just prior to ground contact [56]. There is also evidence that feedback from stretch receptors is vital for maintaining the frequency and duration of regular locomotive movements in some insects [62]. Our controller demonstrates that feedback of hip extreme flexion angle is a suitable and effective means of triggering knee extension at terminal swing, initiating the swing-to-stance transition and ensuring stability of the walker while protecting the mechanical hip joint from overflexion.

Compared to classical control systems used in robotics including, finite-state machines, trajectory tracking, machine learning and CPGs, our controller is based on actual human walking data. We have created a closed-loop system based on the causal relationship between the foot contact information and the muscle activation signals (in humans), which translates to motor activation at the limbs in the robot. The result is so-called limit cycle walking which is defined by Hobbelen and Wisse (2007) as a nominally periodic sequence of steps which although not locally stable at every instant in time, is stable as a whole [63]. Limit cycle walking allows a walker to adapt its gait to the changing natural dynamics producing a convergence to a desired motion following any deviation from the desired trajectory, using only zero or low feedback gains. As can be expected this is more energy efficient than using high feedback gain to force the walker to remain on an intended path, which is a constant fight against natural deviations [63]. Our controller demonstrates limit cycle walking in RunBot as the motion is able to return naturally to the desired trajectory following a disturbance, after only a short time and without CPGs or trajectory control.

The precise function of load dependent reflexes and the extent to which reflex responses generated by sensory input from peripheral receptors contribute to human bipedal gait in comparison to other mammals is not thoroughly understood. It is still unclear how significant spinal networks are in the generation of human walking and whether the functional effect of load receptors and reflexes play a similar role in human muscle activation as in the animal models.

Neurophysiological studies have revealed in different animal species that during locomotion (including walking, flying, swimming etc.), motor neurons are being driven by CPGs. These central networks have been observed to work independent of sensory or descending inputs carrying specific timing information and generate the rhythm and pattern of the locomotor bursts of the motor neurons [64], [65]. CPGs were first successfully demonstrated in the oscillatory output of the deafferented locust wing in response to non-rhythmic stimulation of the nerve cord which was maintained in the complete absence of sensory input [66]–[68]. CPGs have been identified and documented in mammals such as the cat but for humans they have yet to be conclusively described as the experimental procedures used cannot be replicated (for review see [69]). The significant amount of evidence for locomotor CPGs in various different animals suggests it would be very unusual if a similar system was completely absent in humans. However humans are unique among mammals as habitual bipeds making comparison to an animal model difficult. The lack of evidence could be due to other mechanisms being of primary importance such as contribution from reflexive and supraspinal controls. One significant observation highlighting differences between potential human CPGs and those found in other species is that following a complete spinal cord injury, humans become completely paralysed below the level of injury and locomotor activity is typically not evident for many years [70], whereas rhythmic stepping can be evoked in a cat after complete spinal transection ([64] for review). A study of patients with spinal cord injuries (SCI) by Dietz et al. (2002) describes a limited coordination between the legs suggesting the coupling between any CPGs is weak when the input from supraspinal structures is reduced [71]. Similarly, an extensive study on Macaque monkeys with transected spinal cords failed to produce hind leg stepping using procedures similar to those used on cats, which raises doubt over the existence of locomotor CPGs in primates. However, rhythmic alternating activity could be generated if part of the spinal cord was left intact and more successfully when locomotor centres in the brain stem were stimulated in decerebrate animals with an intact spinal cord [70], [72], [73]. The conclusion from primate studies is that if a CPG is present in primates then it is more dependent on intact supraspinal control than is found in the cat [70].

Unlike a CPG, a reflex is a local motor response to a local sensory input. In the locomotion of human and animals, various reflexes act together to control the limbs and their integration contributes to the regulation of the locomotor gait cycle [74]. The concepts that have emerged from walking studies are that reflexes are dependent on task, phase and context and they require modulation using sensory feedback from peripheral afferents in order to function effectively in locomotion where the initial conditions are changing on every step [74]–[78].

Within a human model, feedback on the current status of the walking process is fed back from different sensory organs located locally in muscles and tendons and peripherally from the vestibular and visual systems. At high walking speeds, coordination between the sensory input and motor output needs to act quickly with efficiency and these high dynamic walking demands are currently not possible using existing artificial robotic control systems [15], [18], [20].

We believe that our controller demonstrates that complex behavioural patterns can result from a simple model for locomotion and gait control based on reflexes. An achievement where much of the biological complexity within the true human motor control system has been omitted.

### Implications

A reflexive controller based on human data has implications for locomotor training after spinal cord injury. Functional electrical stimulation (FES) is commonly recruited as a rehabilitation strategy for SCI to exercise and strengthen weakened muscles as well as artificially replace muscle activation that is missing or lacking (for review see [6]). FES uses small electrical currents to directly stimulate peripheral nerves, alpha motor neurons, to cause muscle contraction. For gait rehabilitation, FES is applied to nerves which innervate leg muscles with particular motor functions during the swing and stance gait phases, activating them (artificially) with timing consistent with a normal walking gait cycle [79]–[84]. Research within the last decade has suggested walking function is vastly improved in individuals with incomplete SCI undergoing functional electrical stimulation (FES) therapy [5].

Sophisticated FES devices have been designed to enable patients with SCI to stand, walk and sit but the most common form of commercial stimulator systems available are primarily for correcting drop-foot and for standing in individuals with paraplegia (for review see [85]). The most simple method of control used by stimulator systems, including the Parastep I (Sigmedics, Inc., Fairborn, OH) [86], [87], is open-loop control to provide stimulation pulses to assist in standing or walking by coordinating the activation of muscles. Open-loop involves no direct feedback back to the controller about the actual state of the system and so there are complications in generating accurate control of movement generation using these systems due to difficulty in predicting the correct timing of stimulus, non-linearity of the neuromuscular-skeletal system and inability for modulation during deviations from an ideal gait cycle [6]. Providing sensory feedback from the patient to the FES device should allow improvement in control of the generated movement and produce walking which is more normal than seen with open-loop systems, improving speed and efficiency [88]. Feedback allows a modulation of the stepping by the walking, adapting the gait in compensation for changes within the terrain or environment.

Automatic control was examined by Popovic et al. (2005) as an alternative for push button control, using a pre-programmed multi-channel electrical stimulation system for stroke patients [89]. Stimulation of the Quadriceps, Gastrocnemius, and Tibialis Anterior was applied for support during stance, push-off at terminal stance, and to provide stability at foot contact, as well as during the swing phase. The timing used for stimulation was pre-set to mimic the onset and switching off of muscle activity found in healthy individuals during slow pace walking. Issues with the system involved the timing, which was based on data from from healthy individuals, and hence did not match the timing of voluntary activity in stroke patients. It was also found that patients with stroke, modified their muscle activation when their muscles were stimulated, especially if the stimulation applied to the muscles was not in phase with any voluntary contraction.

Closed-loop control has been studied using two different forms of sensory feedback; biological signals generated by the individual (EMG, ENG or EEG) and signals derived from artificial sensors. Research involving gait event detection have traditionally been based on a single type or an integration of different body-worn sensors typically positioned on the thigh, shank or foot to measure ambulation and have included accelerometers [90], [91], gyroscopes and FSRs [92], and accelerometers and FSRs [93]. Many closed-loop control strategies for FES applications in SCI individuals have been reported in the literature. These fall into categories which include dynamic controllers, finite state controllers and artificial networks (for full review of FES control see [94]). Similar to controllers developed for bipedal robotic walkers, the different controllers applied to FES have issues with computational power. These issues include: (i) high gain requirements for error correction, (ii) complicated algorithms for trajectory control, and (iii) difficulties in implementing the control strategy on a human model (with complications such as latency, muscle spasticity, voluntary control and fatigue). None of these control methods have managed to produce an adaptive gait pattern based on self-stabilising dynamic processes as observed in natural walking, which may explain why open-loop controllers remain the most common in commercial FES systems.

A study by Kojovic et al. (2009) compared the automatic FES control system, proposed by Popovic et al. (2005) [89], with an FES control system using rule based IF-THEN type finite state control and incorporating artificial feedback from force sensing resistors and accelerometers [93]. They found that this alternative provided timing for muscle activation which was in synch with required voluntary movements. Pappas et al. (2001) combined a gyroscope, measuring the angular velocity of the foot, with force sensing resistors (FSRs), to determine toe-off and heel strike which enabled then to detect the swing phase of gait [92]. Their system success rate was above 96% for subjects with impaired walking.

The main difference between the previously discussed control schemes and ours presented here is that our approach uses linear transfer/filter functions which do not require any thresholding. Although bipedal robots featuring finite-state machines can exhibit a stable limit cycle [95], it is well known from behaviour based robotics [96] that systems acting without any thresholds or states are very robust. Our controller uses a filter, which translates linearly the input of the heel contact into a muscle stimulation signal. The only threshold we had to employ was on the hip anterior extreme angle (AEA) to determine the trigger time for knee extension at terminal swing; however this threshold is not critical and could probably be replaced by a soft threshold.

In the future we would like to adapt the simple reflexive control system employed by RunBot into an FES controller for gait rehabilitation, which could assist stepping and promote limit cycle walking in patients with spinal cord injuries.

In summary, the results presented here demonstrate a simple method for controlling walking by establishing the underlying relationship between ground contact information from the feet and muscle activity, which could be of great importance and has significant potential in the development of bipedal robotics and in rehabilitation strategies.

## Supporting Information

Table S1 **The final mean square error result for each subject for each muscle transfer function from the adaptive filtering.** The transfer functions were calculated using the EMG activity recorded from the subject's right leg with heel contact information from both the right and left foot. Transfer functions related to the Lateral Gastrocnemius (LG), Rectus Femoris (RF) and Biceps Femoris (BF) are given, contralateral heel contact are labelled (CH) and ipsilateral heel contact (IH).
(EPS)

Table S2
**Values for the extreme angle of each joint (** 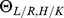 **).** RunBot II features an elastic knee structure so real-time tracking of the knee joint angle is not possible. Instead, the motor position voltage (V) is used to predict the knee joints reaching the joint angle threshold. The hip angles differ from left to right leg due to the effect of RunBot being constrained to a circular walking path and are different values than those documented from humans due to the mechanical structure and the need of RunBot to keep its centre of mass forward.(EPS)

Table S3
**The gain of the motor amplifier (** 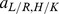 **).**
(EPS)

Video S1 **Experimental set-up.** Video demonstrating the experimental set-up used to record muscle activity (EMG) and foot contact information from the human test subjects.
(MP4)

Video S2 **EMG and foot contact recording during human treadmill walking.**
(MP4)

Video S3 **RunBot walking using transfer function set 1A.**
(MP4)

Video S4 **RunBot walking using transfer function set 1B.**
(MP4)

Video S5 **RunBot walking using transfer function set 2A.**
(MP4)

Video S6 **RunBot walking using transfer function set 2B.**
(MP4)

Video S7 **RunBot walking using transfer function set 3A.**
(MP4)

Video S8 **RunBot walking using transfer function set 3B.**
(MP4)

Video S9 **RunBot walking using transfer function set 4A.**
(MP4)

Video S10 **RunBot walking using transfer function set 4B.**
(MP4)

Video S11 **RunBot walking using transfer function set 5A.**
(MP4)

Video S12 **RunBot walking using transfer function set 5B.**
(MP4)

Video S13 **RunBot walking using transfer function set 6A.**
(MP4)

Video S14 **RunBot walking using transfer function set 6B.**
(MP4)

Data S1 **File information for Data S2 to S21.**
(GZ) Click here for additional data file.

Data S2 **Subject A.**
(GZ) Click here for additional data file.

Data S3 **Subject B.**
(GZ) Click here for additional data file.

Data S4 **Subject C.**
(GZ) Click here for additional data file.

Data S5 **Subject D.**
(GZ) Click here for additional data file.

Data S6 **Subject E.**
(GZ) Click here for additional data file.

Data S7 **Subject F.**
(GZ) Click here for additional data file.

Data S8 **Subject G.**
(GZ) Click here for additional data file.

Data S9 **Subject H.**
(GZ) Click here for additional data file.

Data S10 **Subject I.**
(GZ) Click here for additional data file.

Data S11 **Subject J.**
(GZ) Click here for additional data file.

Data S12 **Subject A.**
(GZ) Click here for additional data file.

Data S13 **Subject B.**
(GZ) Click here for additional data file.

Data S14 **Subject C.**
(GZ) Click here for additional data file.

Data S15 **Subject D.**
(GZ) Click here for additional data file.

Data S16 **Subject E.**
(GZ) Click here for additional data file.

Data S17 **Subject F.**
(GZ) Click here for additional data file.

Data S18 **Subject G.**
(GZ) Click here for additional data file.

Data S19 **Subject H.**
(GZ) Click here for additional data file.

Data S20 **Subject I.**
(GZ) Click here for additional data file.

Data S21 **Subject J.**
(GZ) Click here for additional data file.
