# Correction: Reflex Control of Robotic Gait Using Human Walking Data

**DOI:** 10.1371/journal.pone.0137819

**Published:** 2015-09-03

**Authors:** Catherine A. Macleod, Lin Meng, Bernard A. Conway, Bernd Porr

The following information is missing from the Funding section: Funding was provided by the University of Glasgow (http://www.gla.ac.uk) LKAS scholarship (http://www.gla.ac.uk/services/postgraduateresearch/scholarships/kelvinsmith/). This funding was received by LM.

The complete, correct Funding statement is: Funding was provided by the Engineering and Physical Sciences Research Council (EPSRC) (http://www.epsrc.ac.uk/Pages/default.aspx) through the Doctoral Training Centre (DTC) in Medical Devices, University of Strathclyde (http://www.strath.ac.uk/simd/dtc/). This funding was received by CAM. Funding was also provided by the University of Glasgow (http://www.gla.ac.uk) LKAS scholarship (http://www.gla.ac.uk/services/postgraduateresearch/scholarships/kelvinsmith/). This funding was received by LM. The funders had no role in study design, data collection and analysis, decision to publish, or preparation of the manuscript.
